# Design and Validation of Augmented Reality Stimuli for the Treatment of Cleaning Obsessive-Compulsive Disorder

**DOI:** 10.3389/fpsyg.2021.618874

**Published:** 2021-05-31

**Authors:** Zoilo Emilio García-Batista, Kiero Guerra-Peña, Ivan Alsina-Jurnet, Antonio Cano-Vindel, Luisa Marilia Cantisano-Guzmán, Asha Nazir-Ferreiras, Luciana Sofía Moretti, Leonardo Adrián Medrano, Luis Eduardo Garrido

**Affiliations:** ^1^Escuela de Psicología, Pontificia Universidad Católica Madre y Maestra, Santiago de los Caballeros, Dominican Republic; ^2^Departamento de Personalidad, Evaluación y Tratamientos Psicológicos, Facultad de Psicología, Universidad de Vic-Universitat Central de Catalunya (UVIC-UCC), Barcelona, Spain; ^3^Departamento de Psicología Básica, Facultad de Psicología, Universidad Complutense de Madrid, Spain; ^4^Facultad de Psicología, Universidad Siglo 21, Argentina

**Keywords:** augmented reality, obsessive compulsive disorder, e-health, anxiety, emotion

## Abstract

Fear to contamination is an easy-to-provoke, intense, hard-to-control, and extraordinarily persistent fear. A worsening of preexisting psychiatric disorders was observed during the COVID-19 (coronavirus disease 2019) outbreak, and several studies suggest that those with obsessive–compulsive disorder (OCD) may be more affected than any other group of people. In the face of worsening OCD symptoms, there is a need for mental health professionals to provide the support needed not only to treat patients who still report symptoms, but also to improve relapse prevention. In this line, it is recommended to improve alternative strategies such as online consultations and digital psychiatry. The aim of this study is to develop augmented reality (AR) stimuli that are clinically relevant for patients with cleaning OCD and assess their efficiency to obtain emotionally significant responses. Four AR stimuli were developed: a plastic bag full of garbage, a piece of bread with mold, a dirty sports shoe, and a piece of rotten meat. All stimuli were shown to a clinical group (17 patients with cleaning OCD) and a control group (11 patients without OCD). Relevant results were the design of the AR stimuli. These stimuli were validated with the statistical difference in perceived anxiety in the meat stimuli between the clinical and control groups. Nevertheless, when looking at effect sizes, all stimuli present effect sizes from small (plastic bag) to large (meat), with both shoe and bread between small and medium effect sizes. These results are a valuable support for the clinical use of these AR stimuli in the treatment of cleaning OCD.

## Introduction

Fear to contamination is an easy-to-provoke, intense, hard-to-control, and extraordinarily persistent fear. Besides, it is a kind of fear that is often culturally prescribed, highly spread by the media, which makes it more expansive and contagious (Rachman, 2004; Durna et al., 2019). This fear has been identified as the most common symptom of the obsessive–compulsive disorder (OCD, Mathes et al., 2019). In fact, 46% of OCD patients show obsessions related to fear of contamination even in minor contamination incidents, such as shaking hands or touching dirty objects (Bhikram et al., 2017). Washing or disinfecting hands is the compulsion most associated with contamination obsession (Lozano-Vargas, 2017); people may spend many hours washing and rubbing their hands carefully, which sometimes generates skin bleeding or damage (Jalal et al., 2018).

A worsening of preexisting psychiatric disorders was observed during the coronavirus disease 2019 (COVID-19) outbreak (Yao et al., 2020), and several studies suggest that those with OCD may be more affected than any other group of people (Fineberg et al., 2020). The general fear of infection and the emphasis on handwashing in health advertising lead to worsening OCD symptoms. In this line, Davide et al. (2020) developed a study to assess changes in OCD symptoms during quarantine in OCD patients who had received psychiatric care. The results suggest that the outbreak of COVID-19 generated a worsening of symptoms in patients with symptoms of contamination (compared with patients with other symptoms of OCD) and patients who had not reached a state of remission before quarantine (compared with patients who still showed a state of remission before quarantine).

In the face of worsening OCD symptoms, even in patients who were in a prepandemic state of remission, there is a need for mental health professionals to provide the support needed not only to treat patients who still report symptoms, but also to improve relapse prevention. In this line, it is recommended to improve alternative strategies, such as online consultations and digital psychiatry (Cosić et al., 2020). The COVID-19 outbreak generates the urgent need for integrating technology into innovative models of mental healthcare. However, while the use of new technologies may be useful during a period of isolation, they may also cause a worsening of symptoms if performed without appropriate guidelines (Király et al., 2020).

The use of immersive technologies such as virtual reality (VR) and augmented reality (AR) has proven effective for the treatment of disorders related to anxiety (Carl et al., 2019), thus showing their potential to transform OCD treatment by means of integrating virtual elements (Laforest et al., 2016a; García-Batista et al., 2020). This kind of technologies makes it possible to expose OCD patients to their main fears and dreads in a more controlled, gradual, and safe way (Quero et al., 2017). Different studies have shown that VR allows a high emotional activation in patients with verification and checking (Kim et al., 2008; Van Bennekom et al., 2017), symmetry, and order OCD (Kim et al., 2012; Van Bennekom et al., 2017). However, the use of virtual elements for contamination OCD is incipient (Belloch et al., 2014; Laforest et al., 2016b; García-Batista et al., 2020).

To date, there have been no technological developments that involve the use of AR for the treatment of contamination OCD. This kind of technology allows the addition of digital content to the real world, permitting the visualization of an image formed by the physical environment where they are located (including their own body) with virtual or digital elements overlapped on it [e.g., three-dimensional (3D) objects, texts, sounds, images, or even smells]. This way, and different to VR, AR does not want to replace reality, but improve it, complete it, and expand it with new digital information (Squire and Klopfer, 2007). Besides, AR involves less cost than VR and less technological requirements, thus being simpler to incorporate in the common clinical practice (Eichenberg and Wolters, 2012).

AR could help create a higher level of realism, as during exposure sessions the patients keep seeing the real environment around them while they interact with the virtual stimuli that generate discomfort. These AR features can increase users' sense of presence. The concept of “presence,” most commonly defined as the sense of “being there,” refers to the feeling of being in the virtual world as if it were the real world. Therefore, the sense of presence is rooted on a paradoxical state of consciousness: we behave and feel as if we actually were in the virtual world, even though we know there is nothing there (Iachini et al., 2019). Several studies have documented that the feeling of presence is associated with a greater effect of virtual stimuli (García-Batista et al., 2020). Several factors may induce a high sense of presence, such as the quality of the 3D graphics and the possibility of interaction with the virtual environment (Iachini et al., 2019). For this reason, when developing virtual stimuli, it is important to examine the sense of presence they generate.

This work aims at developing different AR stimuli to treat cleaning OCD and to assess their effectiveness at generating emotional responses. This way, it will be possible to check if the stimuli created are clinically relevant and therefore possible to be used for cleaning-OCD treatment. In particular, we present the following hypotheses: (a) the AR stimuli developed will provoke higher anxiety responses in the OCD group compared to the control group; (b) both groups will experience high levels of virtual presence; and (c) virtual presence will be proportionally associated with the anxiety levels experienced.

## Methods

### Participants

A call for volunteers to participate in a research study related to the use of technological devices for the treatment of cleaning OCD was made through different social networks. Volunteers interested in participating had to go to the Pontificia Universidad Católica Madre y Maestra in the Dominican Republic, where they were given an information sheet with written details about the study and were asked to sign an informed consent form. A total of 28 volunteers participated in the study; 9 of them were men (31%) and 19 were women (69%), aged between 18 and 54 years (mean = 27.11 years, SD = 10.55 years).

Trained team members with more than 10 years of clinical experience administered the questionnaires and a structured interview [Structured Clinical Interview for Axis I Mental Disorder (SCID-I)]. Based on the results obtained from the evaluation, two groups were formed: control group (*n* = 11) and cleaning-OCD group (*n* = 17). The control group was formed by four men (36.4%) and seven women (63.6%), aged between 18 and 50 years (mean = 26.55 years, SD = 9.95 years). Cleaning-OCD group was formed by 5 men (29.4%) and 12 women (70.6%), aged between 19 and 54 years (mean = 27.47 years, SD = 11.19 years). Cleaning-OCD participants are characterized by obtaining a score of more than 13 points (cutoff point) in the Yale–Brown Scale for Obsessive–Compulsive Disorder (Y-BOCS) and by showing contamination/cleaning-OCD symptoms when doing the structured interview (SCID-I).

### Instruments

#### Evaluation

##### SCID-I (First et al., 2002)

It is a semistructured interview that assesses the main groups of Axis I disorders of the DMS-IV-TR, including the OCD. The SCID-I is widely used in mental health, and it offers good psychometric properties (Lobbestael et al., 2011).

##### Symptom Checklist–Revised

Created by Derogatis, it is an instrument to assess a variety of psychological and psychopathological symptoms. It is a 90-item Likert-kind response scale, with five options—from 0 to 4. The evaluation and interpretation are carried out according to nine primary dimensions and three global indexes. On the one side, the dimensions are as follows: somatization, obsessions and compulsions, interpersonal sensitivity, depression, anxiety, hostility, phobic anxiety, paranoid ideation, and psychoticism. On the other, the indexes are as follows: global severity index, positive symptom distress index, and positive symptom total (Derogatis, 1994; Casullo, 1999/2004). Cronbach α coefficient found in the sample of this study was α = 0.97.

##### Y-BOCS

The version adapted to Spanish by Bobes et al. (1994) was used, and specifically, the symptoms related to cleaning OCD. In the Symptom Checklist-17, items were taken into account: the contamination and symmetry/exactness obsessions, and the cleaning/washing and ordering/arranging compulsions. The second part is a Likert scale with a 0- to 4-point score, made up of 10 items that measure the symptomatology degree, evaluating the time obsessive thoughts and compulsions take in their lives, the interference produced, the discomfort related to it, the resistance, and the degree of control over them. The Cronbach α coefficient found in the sample of this study was α = 0.81

##### State-Trait Anxiety Inventory (STAI)

It is an instrument, with a Likert-kind response scale that has two subscales: one assesses anxiety as state (S-A) and the other as trait (T-A). Each subscale has 20 phrases that correspond to the anxiety felt by the person at the moment (S-A) or in general (T-A), and the score goes from 0 to 3 (Buela-Casal et al., 2011). In each subscale, there is a series of phrases whose sense is inverted, aimed at assessing the well-being or lack of anxiety, whereas the others' statements are focused on the presence of anxiety (García-Batista et al., 2018). In the present study, only the anxiety state subscale was used. Psychometric validation studies in Dominican population (García-Batista et al., 2018) indicate optimal levels of internal consistency (α = 0.88).

##### Igroup Presence Questionnaire (IPQ)

This is a self-report questionnaire designed to assess the sense of presence in virtual environments. This scale is formed by 14 items whose responses correspond to a Likert-kind scale with seven response possibilities (Schubert et al., 2001; Igroup, 2019). The IPQ has three subscales that evaluate different dimensions of the sense of presence. Involvement subscale aims at measuring the attention devoted to the virtual environment, the Spatial Presence subscale is related to the sense of being physically part of the simulated environment, and the Realism subscale measures the realism degree granted to the virtual environment. The IPQ also has a general item that assesses the sense of being there. The Cronbach α coefficients found in the sample of this study for each dimension were as follows: α = 0.76 (IPQ-Spatial), α = 0.75 (IPQ-Involvement), α = 0.73 (IPQ-Realness), and α = 0.76 (IPQ-General).

#### Hardware

The virtual environments were developed and executed using an Intel® Core™, i5-6500 CPU @ 3.20 GHz, 3.19-GHz computer, with a RAM memory of 32.0 GB DDR4, and a graphic card of NVIDIA GeForce GTX 1080 (8 GB GDDR5). For this study, low-cost VR headsets were used, such as Google Cardboard 2.0, and a HUAWEI Mate 10 lite smartphone, model: RNE-L03, Android version: 8.0.0, RAM 4.0 GB.

### Procedure

The first step was to carry out a literature review for the design and development of different AR stimuli for the treating of cleaning OCD. We found that the stimuli that generate more anxiety and compulsions in cleaning-OCD patients are those they perceive as objects with germs, virus, fungi, or some other kind of toxic material that can provoke illnesses or cross-contaminations. Among the examples that were mentioned repeatedly in relevant studies are decomposed food (such as rotten meat or food with mold), objects on which dirt can be observed, and objects the person considers can transmit contamination because they were or are in contact with objects, people, or places infected or dirty (Breiter et al., 1996; Rachman, 2004; Armstrong and Olatunji, 2017; Mathes et al., 2019). Based on this, the four aversive stimuli developed in this project were a sealed plastic bag (full of garbage), bread with mold, rotten meat, and sports shoes with dirt on their surface.

After designing and developing the virtual stimuli, the validation study proceeded. Trained team members administered the Y-BOCS and the SCID-I structured interview to identify participants with cleaning-OCD symptoms. In addition, the symptom checklist–revised (SLQ) was administered to screen for the presence of other psychological disorders. Based on the results obtained in that assessment, two groups were formed: the cleaning-OCD group, consisting of participants with scores >13 on the Y-BOCS and who showed contamination/cleaning-OCD symptoms when performing the structured interview (SCID-I), and a control group, including participants with no cleaning-OCD symptomatology and scores <13 on the Y_BOCS.

Once the groups were formed, AR devices were used, presenting the participants with the four stimuli designed for the treatment of cleaning OCD. They were presented in the following order (1) sealed garbage bag, (2) moldy bread, (3) dirty sneakers, and (4) rotten meat. They each presented for 3 min. In the first 2 min, they were to look at the stimulus. They were asked what they could see and described the observed object. The leaf was rotated 90 degrees every 30 s. At the beginning of the last minute, participants were asked to get as close as possible to the stimulus with their hands. After being exposed to each of them, the participant had to complete the STAI (state subscale), and once exposed to the four stimuli, the IPQ.

### Statistical Analyses

To determine if the AR stimuli provoked different levels of anxiety for the clinical and control groups, and if these interacted with the different stimuli presented, we conducted a robust heteroscedastic mixed analysis of variance (ANOVA) based on 20% trimmed means (Mair and Wilcox, 2020). For the mixed ANOVA, the between-subjects factor was the group variable (clinical or control), and the within-subjects factor was the stimuli (bag, bread, shoes, and meat). Additionally, to compare the mean scores in virtual presence between the clinical and control groups, we employed Yuen's two-sample robust heteroscedastic test based on 20% trimmed means (Mair and Wilcox, 2020). Additionally, as a robust measure of standardized effect size, we used the Xi explanatory measure (ξ), which is analogous to a correlation coefficient and can be interpreted using Cohen's guidelines of 10, 0.30, and 0.50, for small, medium, and large effects, respectively. This measure of effect size was also used to estimate the effect size for the between-group effect of the mixed ANOVA.

In order to estimate the level of association between the anxiety responses to the stimuli and the virtual presence scales we employed, the Spearman robust correlation coefficient was used, which is based on rank scores (Bishara and Hittner, 2015, 2017). Similarly, Spearman correlation coefficient was used to estimate the level of association between the anxiety responses and the clinical scales. According to Cohen (1992), correlation levels of 0.10, 0.30, and 0.50 can be considered as small, medium, and large, respectively.

Data handling, descriptive statistics, and Spearman correlation coefficients were computed using the statistical software SPSS, version 25. Robust heteroscedastic mean comparisons based on trimmed means were conducted in R using the package *WRS2* version 1.1-0 (Mair and Wilcox, 2020). Specifically, the robust heteroscedastic mixed ANOVA was computed using the *bwtrim* function and the robust heteroscedastic two-sample test using the *yuen* function. The effect size measures for the between group effects were computed using the *yuen.effect.ci* function.

## Results

### AR Stimuli Development

From the results found in the literature review four AR stimuli were developed (Breiter et al., 1996; Rachman, 2004; Armstrong and Olatunji, 2017; Mathes et al., 2019). These stimuli aimed at causing the emotions experienced by the people who suffer from cleaning OCD. The tridimensional models were created using Autodesk 3D Max. After creating them, UV maps were made to texturize them and export them to Unity 3D. Finally, the Vuforia program was used to develop the AR experience.

The stimuli mentioned were a garbage bag (Breiter et al., 1996), a loaf of bread with mold (Armstrong and Olatunji, 2017), a dirty sports shoe (Mathes et al., 2019), and a piece of meat in decomposition (Rachman, 2004). The app makes it possible to present each stimulus by means of a tablet or low-cost VR headsets such as Google Cardboard 2.0, to facilitate personalized exposure. In this sense, it is worth pointing out that only the Google Cardboard 2.0 glasses were used for this study.

**Figure 1:**
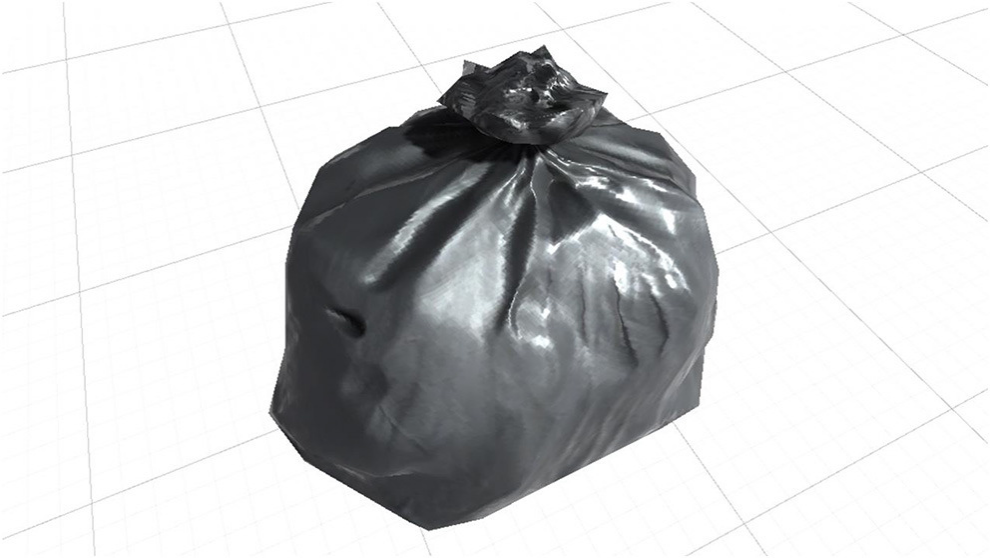
Augmented reality plastic bag.

Next, there is a brief description of the AR stimuli developed:

**Plastic bag** (Figure 1). It is a black bag, the type commonly used to collect garbage. The garbage bag tends to be one of the most aversive stimuli for cleaning-OCD patients (Breiter et al., 1996).

**Figure 2:**
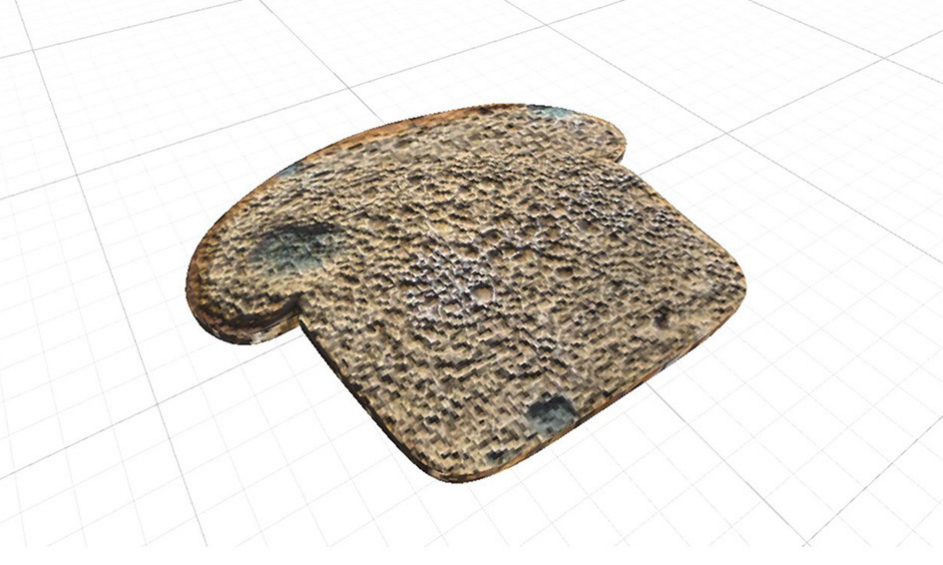
Bread with mold.

**Contaminated food** (Figure 2). It is a loaf of bread clearly contaminated with mold. Its main goal is to generate the anxiety people with cleaning OCD tend to experience when exposed to contaminated food (Armstrong and Olatunji, 2017).

**Figure 3:**
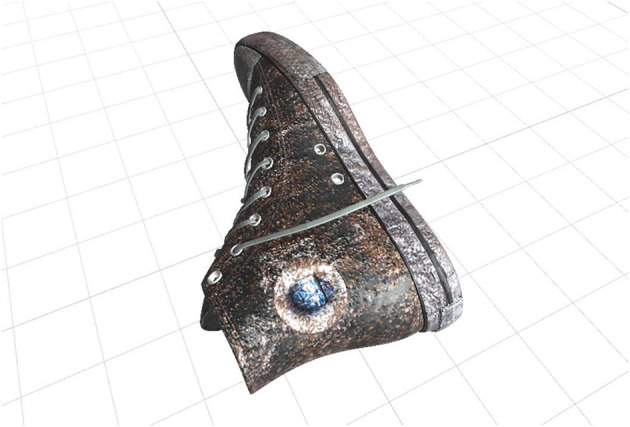
Dirty sports shoe.

**Dirty sports shoe** (Figure 3). It is a shoe with mud and other dirt details. Its goal is to induce the anxiety people with cleaning OCD often experience when exposed to objects they perceive as dirty (Mathes et al., 2019).

**Figure 4:**
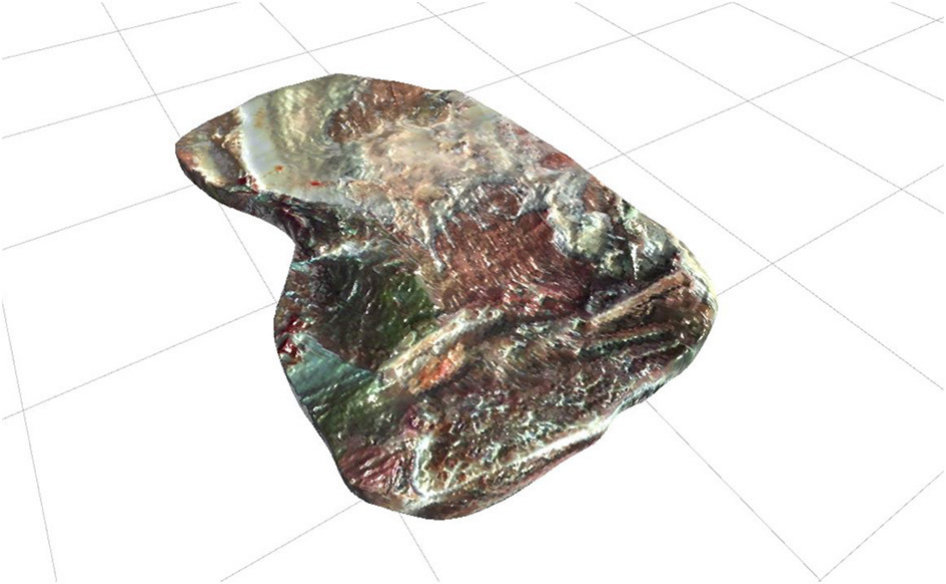
Rotten meat.

**Rotten meat** (Figure 4). It is a highly rotten piece of meat. Its aim is to generate the anxiety people with cleaning OCD tend to experience when exposed to rotten meat or decaying food (Rachman, 2004).

### Assessment of AR Stimuli Effectiveness: Anxiety Response

**Figure 5:**
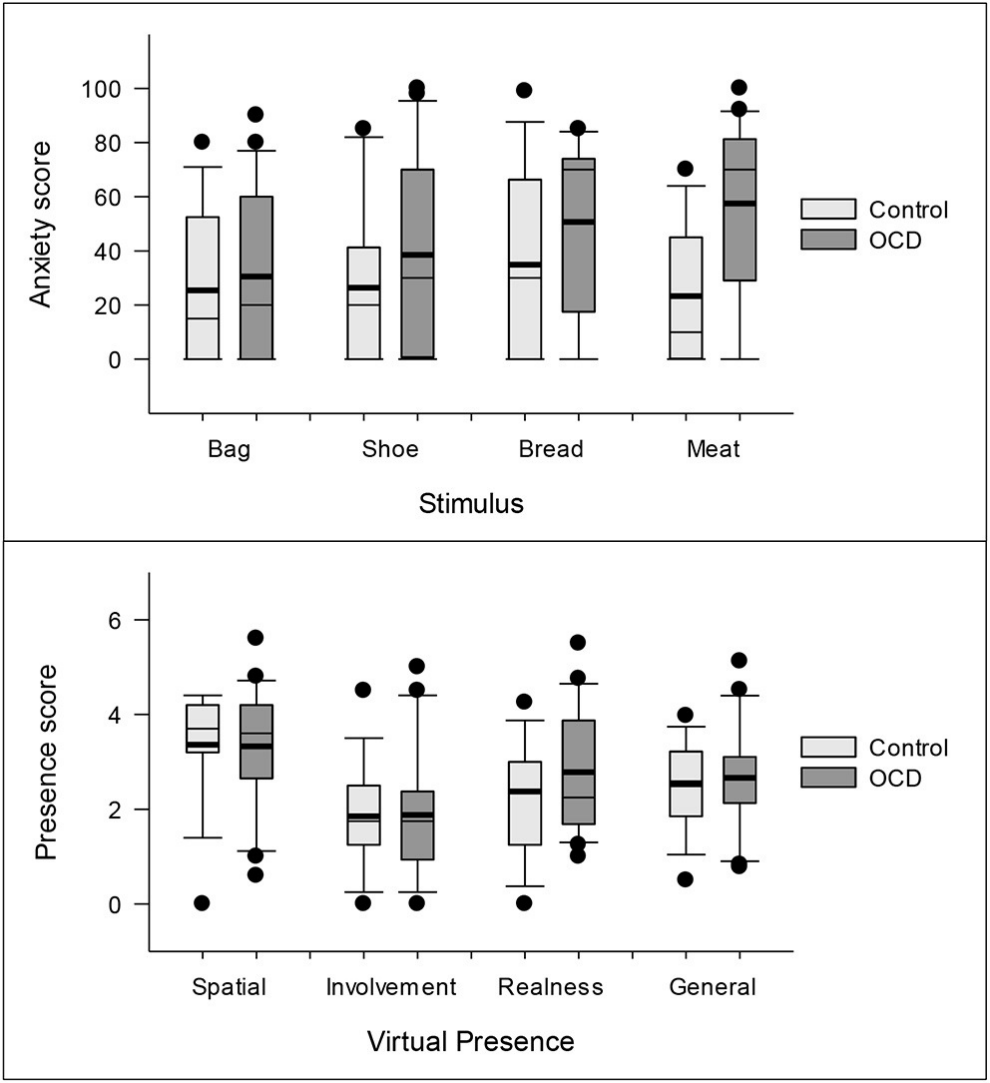
Box plots for the anxiety and virtual presence scores across the control and clinical groups. The thin lines inside the boxes represent the median and the thick ones, the means. The circles represent the observations outside the 10–90th percentiles.

The descriptive statistics for the anxiety responses to the AR stimuli are shown in Table 1 (upper section). These include the means (M), standard deviations (SDs), 20% trimmed means (Mt), and standard errors for the 20% trimmed means (Mt.se). Additionally, the distribution of the anxiety response variables is represented in Figure 5 (upper panel) via box plots.

**Table 1:** Descriptive statistics for the anxiety and virtual presence scores.

	**Total (*****n*** **= 28)**				**Control (*****n*** **= 11)**				**Clinical (*****n*** **= 17)**			
**Variables**	**M**	**SD**	**Mt**	**Mt.se**	**M**	**SD**	**Mt**	**Mt.se**	**M**	**SD**	**Mt**	**Mt.se**
*Anxiety*												
Bag	28.54	31.26	23.28	8.44	25.45	30.20	19.29	13.21	30.53	30.20	25.82	11.23
Shoe	33.79	34.44	27.67	9.06	26.36	31.95	17.86	10.14	38.59	31.95	33.91	11.96
Bread	44.50	33.84	45.39	10.22	34.91	35.95	29.29	15.83	50.71	35.95	55.64	11.49
Meat	44.04	36.19	43.67	10.71	23.27	26.40	18.00	10.99	57.47	26.40	63.18	13.60
Overall	37.71	34.21	34.26	4.90	27.50	30.53	20.57	6.16	44.32	35.04	44.38	6.66
*V. presence*												
Spatial	3.34	1.30	3.60	0.19	3.36	1.30	3.67	0.24	3.33	1.34	3.51	0.33
Involvement	1.87	1.37	1.69	0.23	1.85	1.24	1.83	0.29	1.88	1.48	1.64	0.33
Realness	2.63	1.33	2.57	0.29	2.38	1.33	2.54	0.44	2.78	1.34	2.64	0.45
General	2.61	1.09	2.63	0.17	2.53	1.02	2.62	0.31	2.66	1.16	2.63	0.20

*n, sample size; M, mean; SD, standard deviation; Mt, 20% trimmed mean; Mt.se, standard error for the 20% trimmed mean; V. presence, virtual presence*.

**Figure 6:**
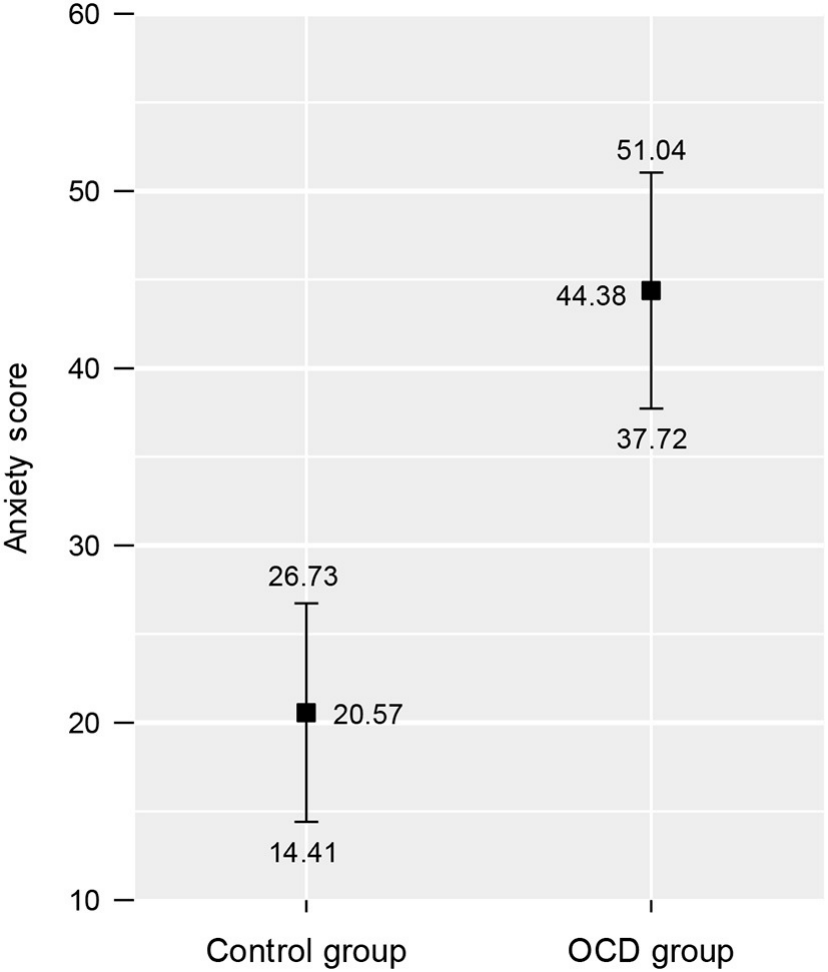
Overall mean anxiety scores across the augmented reality stimuli for the control and clinical groups. The squares represent the 20% trimmed means, and the intervals show the standard errors of the 20% trimmed means.

A robust heteroscedastic mixed ANOVA based on 20% trimmed means showed that the four AR stimuli (bag, shoes, bread, and meat) did not produce significantly different mean levels of anxiety on the participants (*F*_3, 16.46_ = 1.31, *p* = 0.305). Conversely, the mixed ANOVA showed that the clinical group (Mt = 44.38, Mt.se = 6.66) experienced higher anxiety levels than the control group (Mt = 20.57, Mt.se = 6.16) in response to the AR stimuli (*F*_1, 22.28_ = 5.95, *p* = 0.023). According to the robust measure of effect size, these differences between the clinical and control groups could be categorized as being medium sized (ξ = 0.37). The overall anxiety trimmed means and their standard errors for the control and clinical groups are presented in Figure 6. Finally, the mixed ANOVA showed that there was *no* interaction in the anxiety scores between group membership (control vs. clinical) and the AR stimuli (*F*_3, 15.68_ = 0.73, *p* = 0.548).

### Assessment of AR Stimuli Effectiveness: Virtual Presence

In order to compare the means of the virtual presence scale scores between the clinical and control groups, we employed Yuen's two-sample robust heteroscedastic test based on 20% trimmed means. The results indicated that none of the virtual presence scores, spatial (*t*_14.76_ = 0.40, *p* = 0.697), involvement (*t*_13.46_ = 0.45, *p* = 0.657), realness (*t*_12.55_ = 0.15, *p* = 0.881), and general (*t*_8.69_ = 0.03, *p* = 0.978), were different between the two groups. As there was no difference in the moderate scores between the groups, the means values were interpreted for the complete group. In every case, the possible value range was between 0 and 6, where 3 was the midpoint of the scale, and higher scores showed higher virtual presence. Thus, the spatial presence scale means exceeded the midpoint of the scale (mean = 3.34, SD = 1.30), realness got close to the midpoint (mean = 2.63, SD = 1.33), and involvement obtained the lowest level (mean = 1.87, SD = 1.37).

The final aim was to determine if the virtual presence was related to the anxiety responses to the stimuli. This aim was assessed by means of the Spearman correlation coefficient between the variables (Table 2). In terms of the correlations, the results in Table 2 indicate that the involvement, realness, and general presence scales had medium to high significant positive correlations with the anxiety responses to the virtual stimuli. Specifically, involvement had medium to high correlations with the anxiety provoked by the bag (*r*_s_ = 0.435, *p* < 0.05), the shoe (*r*_s_ = 0.536, *p* < 0.01), and the bread (*r*_s_ = 0.544, *p* < 0.01). Similarly, realness had medium correlations with the anxiety provoked by the shoe (*r*_s_ = 0.404, *p* < 0.05) and the bread (*r*_s_ = 0.459, *p* < 0.05). Finally, general presence obtained medium to high positive correlations with the anxiety provoked by the bag (*r*_s_ = 0.458, *p* < 0.05), the shoe (*r*_s_ = 0.495, *p* < 0.01), and the bread (*r*_s_ = 0.532, *p* < 0.01).

**Table 2:** Spearman correlations between the anxiety and virtual presence variables.

**Variable**	**1**	**2**	**3**	**4**	**5**	**6**	**7**
1. Bag anxiety	—						
2. Shoe anxiety	0.506^**^	—					
3. Bread anxiety	0.587^**^	0.743^**^	—				
4. Meat anxiety	0.729^**^	0.543^**^	0.591^**^	—			
5. IPQ-Spatial	0.254	0.252	0.316	0.177	—		
6. IPQ-Involvement	0.435^*^	0.536^**^	0.544^**^	0.337	0.474^*^	—	
7. IPQ-Realness	0.366	0.404^*^	0.459^*^	0.339	0.587^**^	0.370	—
8. IPQ-General	0.458^*^	0.495^**^	0.532^**^	0.364	0.819^**^	0.691^**^	0.861^**^

*IPQ, Igroup Presence Questionnaire*.

* *p < 0.05*,

** *p < 0.01*.

## Discussion

OCD can be a highly weakening condition associated with considerable distress that deteriorates significantly the quality of life of those who suffer from it. Cleaning OCD affects 46% of the OCD patients and is characterized by serious contamination fears and excessive washing habits. This kind of OCD damages their physical health (e.g., it may provoke dermatitis due to the excessive use of cleaning products), their mental health (as comorbidity can occur with anxiety or depression), and their social health (promoting isolation, interfering with their working and academic performance, etc.) (Veale and Roberts, 2014; Torres et al., 2016; Lozano-Vargas, 2017). A worsening of preexisting psychiatric disorders was observed during the COVID-19 outbreak (Yao et al., 2020), and several studies suggest that those with OCD may be more affected than any other group of people (Fineberg et al., 2020).

The top-notch non-pharmacological treatment for this kind of OCD is “exposure and response prevention” (Abramowitz et al., 2001; Franklin and Foa, 2002; Vallejo, 2003). This kind of intervention is the repeated, prolonged, and systematic exposure to situations and thoughts that generate discomfort while preventing manifest or hidden compulsions. Despite being the most used treatment, up to 40% of patients with OCD do not respond to the treatment (Rosa Alcázar and Rodríguez Olivares, 2010; Jalal et al., 2018). Therefore, new non-pharmacological treatments that allow a more effective approach are required. The use of immersive technologies such as AR allows for the improvement of OCD treatment by integrating virtual elements (Laforest et al., 2016a; García-Batista et al., 2020). This work had two main goals: (a) to develop AR stimuli for OCD treatment and (b) to assess the effectiveness of said stimuli by considering the anxiety and virtual presence responses.

Based on the literature revision (Laforest et al., 2016b; García-Batista et al., 2020), four AR stimuli were developed: a garbage bag, a dirty sports shoe, a loaf of bread with mold, and rotten meat. The results obtained agree with what was reported in previous researches regarding the efficiency of the AR stimuli to induce anxiety responses (Bretón-López et al., 2010; Acar et al., 2014). The results of this study show that the means of the state anxiety levels for all the stimuli were higher for the clinical group than for the control one. Despite this, the only significant differences were with the rotten meat. The lack of significant differences in the rest of the stimuli is probably due to the reduced size of this study's sample. However, when considering the sizes of the effect, the differences in the anxiety levels between the clinical and the control groups were moderate and strong. It is also worth pointing out that the stimuli created tend to generate certain aversion level in the general population, which may have diminished the differences between the two groups.

It is also necessary to highlight the differences and similarities found in relation to the anxiety levels generated by each stimulus. In this sense, it was found that the moderate anxiety levels provoked with the bread with mold and the rotten meat were higher than the ones induced by the garbage bag and the shoe. These results can be related to the fact that taste is the sense that most relates with disgust, which is the most sensitized emotion in patients with OCD and which can generate anxiety (Pineda et al., 2015; Bhikram et al., 2017; Knowles et al., 2019). These findings agree with previous studies that show that edible contaminated stimuli generate more disgust and anxiety responses than non-edible ones (Vicario et al., 2017).

Besides, the hypothesis related to the virtual presence of AR stimuli was verified. In concrete, it is observed that both groups get high levels of presence and that higher levels of presence are related to higher anxiety responses. This result reinforces the idea that VR's and AR's efficiency as exposure techniques lies in the possibility of inducing a high level of presence in the user (Wiederhold and Wiederhold, 2005; Alsina-Jurnet et al., 2011; Cummings and Bailenson, 2016; Freeman et al., 2017). In fact, there was a significant correlation between the emotional activation and the attention degree (involvement) and the spatial presence.

Despite some limitations, such as the small sample size, the results obtained are promising and suggest that the use of the designed stimuli may help to apply the exposure and response prevention technique in the therapy, appropriately monitored by the therapist (Eichenberg and Wolters, 2012; Bouchard et al., 2017; García-Batista et al., 2020). Nevertheless, these findings should be confirmed by new studies in wider clinical samples and combining the use of self-report questionnaires with anxiety physiological measures. Also, new AR stimuli should be developed, and evidence of external validity should be provided, i.e., whether habituation to AR stimuli might generalize to real-life stimuli and hence translate to clinical improvement.

It is worth mentioning that this work is pioneer in proposing the use of emerging technologies such as AR for OCD treatment. With this work, specialists have another technological tool for OCD treatment, a highly complex and treatment-resistant disorder (Jalal et al., 2018).

Likewise, it is timely to pinpoint that this is the first time this kind of technology is developed in a Latin American context. The development of this kind of tools, cheaper than VR, would permit the improvement of the deficient access to mental health that prevails in some Latin American countries such as the Dominican Republic. In fact, the use of the AR stimuli developed in this work would allow therapists to apply the “exposure and response prevention” treatment in cleaning-OCD patients, thus creating a positive impact in the psychological well-being and in the quality of life of those who suffer from this disorder.

## Data Availability Statement

The raw data supporting the conclusions of this article will be made available by the authors, without undue reservation.

## Ethics Statement

The studies involving human participants were reviewed and approved by Comité Nacional de Bioetica en Salud/Protocol Number 028-2014. The patients/participants provided their written informed consent to participate in this study.

## Author Contributions

All authors listed have made a substantial, direct and intellectual contribution to the work, and approved it for publication.

## Conflict of Interest

The authors declare that the research was conducted in the absence of any commercial or financial relationships that could be construed as a potential conflict of interest.

## References

[B1] Abramowitz J. S. Brigidi B. D. Roche K. R. (2001). Cognitive-behavioral therapy for obsessive-compulsive disorder: a review of the treatment literature. Res. Soc. Work Pract. 11, 357–372. 10.1177/104973150101100305

[B2] Acar D. Miman M. Akirmak O. O. (2014). Treatment of anxiety disorders patients through EEG and Augmented Reality. Eur. Soc. Sci. Res. J. 3, 18–27.

[B3] Alsina-Jurnet I. Gutiérrez-Maldonado J. Rangel-Gómez M. V. (2011). The role of presence in the level of anxiety experienced in clinical virtual environments. Comput. Hum. Behav. 27, 504–512. 10.1016/j.chb.2010.09.018

[B4] Armstrong T. Olatunji B. O. (2017). Pavlovian disgust conditioning as a model for contamination-based OCD: evidence from an analogue study. Behav. Res. Ther. 93, 78–87. 10.1016/j.brat.2017.03.009 28391115 PMC5540341

[B5] Belloch A. Cabedo E. Carrió A. Lozano-Quilis J. A. Gil-Gómez J. A. Gil-Gómez H. (2014). Exposición mediante realidad virtual para el TOC: ¿Es factible? Revista de Psicopatología y Psicología Clínica 19, 37–44. 10.5944/rppc.vol.19.num.1.2014.12981

[B6] Bhikram T. Abi-Jaoude E. Sandor P. (2017). OCD: obsessive-compulsive … disgust? The role of disgust in obsessive-compulsive disorder. J. Psych. Neurosci. 42, 300–306. 10.1503/jpn.160079 28375077 PMC5573572

[B7] Bishara A. J. Hittner J. B. (2015). Reducing bias and error in the correlation coefficient due to nonnormality. Educ. Psychol. Meas. 75, 785–804. 10.1177/0013164414557639 29795841 PMC5965513

[B8] Bishara A. J. Hittner J. B. (2017). Confidence intervals for correlations when data are not normal. Behav. Res. Methods 49, 294–309. 10.3758/s13428-016-0702-8 26822671

[B9] Bobes J. Bousoño M. González M. P. (1994). Manejo de los trastornos mentales y del comportamiento en Asistencia Primaria. Oviedo: Imprenta Gofer.

[B10] Bouchard S. Dumoulin S. Robillard G. Guitard T. Klinger E. Forget H.. (2017). Virtual reality compared with in vivo exposure in the treatment of social anxiety disorder: a three-arm randomised controlled trial. Br. J. Psychiatry 210, 276–283. 10.1192/bjp.bp.116.184234 27979818

[B11] Breiter H. C. Rauch S. L. Kwong K. K. Baker J. R. Weisskoff R. M. Kennedy D. N.. (1996). Functional magnetic resonance imaging of symptom provocation in obsessive-compulsive disorder. Arch. Gen. Psychiatry 53, 595–606. 10.1001/archpsyc.1996.01830070041008 8660126

[B12] Bretón-López J. Quero S. Botella C. García-Palacios A. Baños R. M. Alcañiz M. (2010). An augmented reality system validation for the treatment of cockroach phobia. Cyberpsychol. Behav. Soc. Netw. 13, 705–710. 10.1089/cyber.2009.0170 21142997

[B13] Buela-Casal G. Guillén-Riquelme A. Seisdedos N. (2011). STAI: Cuestionario de ansiedad estado-rasgo. Adaptación española. Madrid: TEA Ediciones.

[B14] Carl E. Stein A. T. Levihn-Coon A. Pogue J. R. Rothbaum B. Emmelkamp P.. (2019). Virtual reality exposure therapy for anxiety and related disorders: a meta-analysis of randomized controlled trials. J. Anxiety Disord. 61, 27–36. 10.1016/j.janxdis.2018.08.003 30287083

[B15] Casullo M. M. (1999/2004). El inventario de síntomas SCL-90-R de L. Derogatis. Available online at: https://docplayer.es/5996340-El-inventario-de-sintomas-scl-90-r-de-l-derogatis-maria-martina-casullo-1999-2004.html (accessed April 29, 2021).

[B16] Cohen J. (1992). A power primer. Psychol. Bull. 112, 155–159. 10.1037/0033-2909.112.1.155 19565683

[B17] Cosić K. Popović S. Šarlija M. KesedŽić I. (2020). Impact of human disasters and Covid-19 pandemic on mental health: potential of digital psychiatry. Psychiatr. Danubina 32, 25–31. 10.24869/psyd.2020.25 32303026

[B18] Cummings J. J. Bailenson J. N. (2016). How immersive is enough? A meta-analysis of the effect of immersive technology on user presence. Media Psychol. 19, 272–309. 10.1080/15213269.2015.1015740

[B19] Davide P. Andrea P. Martina O. Andrea E. Davide D. Mario A. (2020). The impact of the COVID-19 pandemic on patients with OCD: effects of contamination symptoms and remission state before the quarantine in a preliminary naturalistic study. Psychiatry Res. 291:113213. 10.1016/j.psychres.2020.113213 32535508 PMC7280119

[B20] Derogatis L. (1994). SCL-90- R. Adaptación castellana de la técnica. Facultad de Psicología. Universidad de Buenos Aires. Manual. Minnesota: National Computer Systems.

[B21] Durna G Yorulmaz O Aktaç A. (2019). Public stigma of obsessive compulsive disorder and schizophrenic disorder: is there really any difference? Psychiatry Res. 271:559–564. 30554103 10.1016/j.psychres.2018.12.065

[B22] Eichenberg C. Wolters C. (2012). Virtual Realities in the Treatment of Mental Disorders: A Review of the Current State of Research. InTech.

[B23] Fineberg N. A. Van Ameringen M. Drummond L. Hollander E. Stein D. J. Geller D.. (2020). How to manage obsessive-compulsive disorder (OCD) under COVID-19: A clinician's guide from the International College of Obsessive Compulsive Spectrum Disorders (ICOCS) and the Obsessive-Compulsive Research Network (OCRN) of the European College of Neuropsychopharmacology. Compr. Psychiatry 100:152174. 10.1016/j.comppsych.2020.152174 32388123 PMC7152877

[B24] First M. B. Spitzer R. L. Gibbon M. Williams J. B. W. (2002). Structured Clinical Interview for DSM-IV-TR Axis I Disorders, Research Version, Non-patient Edition (SCIDI/NP). Nueva York: Biometrics Research, New York State Psychiatric Institute.

[B25] Franklin M. E. Foa E. B. (2002). Cognitive behavioral treatments for obsessive compulsive disorder, in A Guide to Treatments That Work, 2nd Edn, eds Nathan P. Gorman J. (Nueva York: Oxford University Press), 367–386.

[B26] Freeman D. Reeve S. Robinson A. Ehlers A. Clark D. Spanlang B.. (2017). Virtual reality in the assessment, understanding, and treatment of mental health disorders. Psychol. Med. 47, 2393–2400. 10.1017/S003329171700040X 28325167 PMC5964457

[B27] García-Batista Z. E. Guerra-Peña K. Alsina-Jurnet I. Cano-Vindel A. Herrera Martínez S. X. Jiménez-Payano D.. (2020). Design of virtual environments for the treatment of agoraphobia: inclusion of culturally relevant elements for the population of the Dominican Republic. Comput. Hum. Behav. 102, 97–102. 10.1016/j.chb.2019.08.015

[B28] García-Batista Z. E. Guerra-Peña K. Cano-Vindel A. Herrera-Martínez S. X. Medrano L. A. (2018). Validity and reliability of the Beck Depression Inventory (BDI-II) in general and hospital population of Dominican Republic. PLoS ONE 13:e0199750. 10.1371/journal.pone.0199750 29958268 PMC6025862

[B29] Iachini T. Ruggiero G. Bartolo A. Rapuano M. Ruotolo F. (2019). The effect of body-related stimuli on mental rotation in children, young and elderly adults. Sci. Rep. 9:1169. 10.1038/s41598-018-37729-7 30718610 PMC6362092

[B30] Igroup (2019). Igroup presence questionnaire (IPQ) overview. Available online at: http://www.igroup.org/pq/ipq/index.php (accessed October 9, 2019).

[B31] Jalal B. Brühl A. O'Callaghan C. Piercy T. Cardinal R. N. Ramachandran V. S.. (2018). Novel smartphone interventions improve cognitive flexibility and obsessive-compulsive disorder symptoms in individuals with contamination fears. Sci. Rep. 8:14923. 10.1038/s41598-018-33142-2 30353111 PMC6199277

[B32] Kim K. Kim C.-H. Cha K. R. Park J. Han K. Kim Y. K.. (2008). Anxiety provocation and measurement using virtual reality in patients with obsessive-compulsive disorder. CyberPsychol. Behav. 11, 637-641. 10.1089/cpb.2008.0003 18991527

[B33] Kim K. Roh D. Kim S. I. Kim C.-H. (2012). Provoked arrangement symptoms in obsessive–compulsive disorder using a virtual environment: a preliminary report. Comput. Biol. Med. 42, 422–427. 10.1016/j.compbiomed.2011.12.010 22226644

[B34] Király O. Potenza M. N. Stein D. J. King D. L. Hodgins D. C. Saunders J. B.. (2020). Preventing problematic internet use during the COVID-19 pandemic: consensus guidance. Compr. Psychiatry 100:152180. 10.1016/j.comppsych.2020.152180 32422427 PMC7215166

[B35] Knowles K. A. Jessup S. C. Olatunji B. O. (2019). Disgust in anxiety and obsessive-compulsive disorders: recent findings and future directions. Curr. Psychiatry Rep. 20:68. 10.1007/s11920-018-0936-5 30094516 PMC6422162

[B36] Laforest M. Bouchard S. Bossé J. Mesly O. (2016a). Effectiveness of in virtuo exposure and response prevention treatment using cognitive–behavioral therapy for obsessive–compulsive disorder: a study based on a single-case study protocol. Public Ment. Health 7:99. 10.3389/fpsyt.2016.00099 27378951 PMC4904031

[B37] Laforest M. Bouchard S. Crétu A.-M. Mesly O. (2016b). Inducing an anxiety response using a contaminated virtual environment: validation of a therapeutic tool for obsessive–compulsive disorder. Front. ICT 3:18. 10.3389/fict.2016.00018

[B38] Lobbestael J. Leurgans M. Arntz A. (2011). Inter-rater reliability of the structured clinical interview for DSM-IV Axis I Disorders (SCID I) and Axis II Disorders (SCID II). Clin. Psychol. Psychotherapy 18, 75–79. 10.1002/cpp.693 20309842

[B39] Lozano-Vargas A. (2017). Aspectos clínicos del trastorno obsesivo-compulsivo y trastornos relacionados. Revista de neuro-psiquiatría 80, 35–41. 10.20453/rnp.v80i1.3058

[B40] Mair P. Wilcox R. (2020). Robust statistical methods in R using the WRS2 package. Behav. Res. Methods 52, 464–488. 10.3758/s13428-019-01246-w 31152384

[B41] Mathes B. M. McDermott K. A. Okey S. A. Vazquez A. Harvey A. M Cougle J. R. (2019). Mental contamination in Obsessive-Compulsive Disorder: associations with contamination symptoms and treatment response. Behav. Ther. 50, 15–24. 10.1016/j.beth.2018.03.005 30661555

[B42] Pineda D. Villaescusa-Alejo V. Sandín B. (2015). Relación entre propensión, sensibilidad al asco y selección de rama profesional. Acción Psicológica 12, 31–42. 10.5944/ap.12.2.10774

[B43] Quero S. Andreu-Mateu S. Moragrega I. Baños R. Molés M. Nebot S.. (2017). Un programa cognitivo-conductual que utiliza la realidad virtual para el tratamiento de los trastornos adaptativos: Una serie de casos. Rev. Argentina Clín. Psicológica 26, 5–18.

[B44] Rachman S. (2004). Fear of contamination. Behav. Res. Ther. 42, 1227–1255. 10.1016/j.brat.2003.10.009 15381436

[B45] Rosa Alcázar A. I. Rodríguez Olivares J. (2010). El trastorno obsesivo-compulsivo en niños y adolescentes. Madrid: Pirámide, 15–243.

[B46] Schubert T. Friedmann F. Regenbrecht H. (2001). The experience of presence: factor analytic insights. Presence 10, 266–281. 10.1162/105474601300343603

[B47] Squire K. Klopfer E. (2007). Augmented reality simulations on handheld computers. J. Learn. Sci. 16, 371–413. 10.1080/10508400701413435

[B48] Torres A. R. Fontenelle L. F. Shavittc R. G. Ferrão Y. A. Rosário M. C. Storch E. A.. (2016). Comorbidity variation in patients with obsessive–compulsive disorder according to symptom dimensions: results from a large multicentre clinical sample. J. Affect. Disord. 190, 508–516. 10.1016/j.jad.2015.10.051 26561941

[B49] Vallejo M. A. (2003). Guía de tratamientos psicológicos eficaces para el trastorno obsesivo compulsivo, in Guía de tratamientos psicológicos eficaces I: Adultos, eds Pérez M. Fernández J.R. Fernández C. Amigo I. (Madrid: Pirámide), 337–353.

[B50] Van Bennekom M. J. Kasanmoentalib M. S. de Koning P. P. Dennys D. (2017). A virtual reality game to assess obsessive-compulsive disorder. Cyberpsychol. Behav. Soc. Netw. 20, 718–722. 10.1089/cyber.2017.0107 29125791

[B51] Veale D. Roberts A. (2014). Obsessive-compulsive disorder. Br. Med. J. 348:g2183. 10.1136/bmj.g2183 24709802

[B52] Vicario C. M. Rafal R. D. Borgomaneri S. Paracampo R. Kritikos A. Avenanti A. (2017). Pictures of disgusting foods and disgusted facial expressions suppress the tongue motor cortex. Social Cogn. Affect. Neurosci. 12, 352–362. 10.1093/scan/nsw129 27614770 PMC5390717

[B53] Wiederhold B. K. Wiederhold M. D. (2005). The effect of presence on virtual reality treatment, in Virtual Reality Therapy for Anxiety Disorders: Advances in Evaluation and Treatment, eds Wiederhold B. K. Wiederhold M. D. (Washington, DC: American Psychological Association), 77–86. 10.1037/10858-006

[B54] Yao H. Chen J. H. Xu Y. F. (2020). Patients with mental health disorders in the COVID-19 epidemic. Lancet Psychiatry 7:e21. 10.1016/S2215-0366(20)30090-0 32199510 PMC7269717

